# Phenotypic and functional characteristics of IL-21-expressing CD8^+^ T cells in human nasal polyps

**DOI:** 10.1038/srep30362

**Published:** 2016-07-29

**Authors:** Li Xiao, Lei Jia, Lu Bai, Long He, Binyan Yang, Changyou Wu, Huabin Li

**Affiliations:** 1 Institute of Immunology, Zhongshan School of Medicine, Key Laboratory of Tropical Disease Control Research of Ministry of Education, Sun Yat-sen University, Guangzhou, China; 2 Allergy Center, Otorhinolarygology Hospital, The First Affiliated Hospital of Sun Yat-sen University, Guangzhou, China; 3 Department of Otolaryngology, Guangdong General Hospital, Guangzhou, China; 4 Department of Otolaryngology, Guangzhou First People’s Hospital, Guangzhou Medical University, Guangzhou, China; 5 Shanghai Key Laboratory of Translational Medicine on Ear and Nose Diseases, Department of Otolaryngology, Head and Neck Surgery, Xinhua Hospital, Shanghai Jiaotong University School of Medicine, Shanghai, China

## Abstract

Although CD4^+^ T cells are recognized to play an important role in the inflammatory response of nasal polyps (NPs), the biological functions of CD8^+^ T cells in polypogenesis remain unclear. In this study, we analyzed cell markers, cytokine expression and transcription factors in IL-21-expressing CD8^+^ T cells in polyp tissues of NP patients. The results showed that the majority of IL-21-producing CD8^+^ T cells were effector memory cells and they co-expressed IFN-γ. IL-21-expressing CD8^+^ T cells in polyp tissues expressed higher CXCR5, PD-1, and ICOS levels than cells in control tissues and showed significantly higher T-bet and Bcl-6 expression levels compared with IL-21^−^CD8^+^ T cells. Purified polyp CD8^+^ T cells promoted IgG production from isolated polyp B cells *in vitro*, and recombinant IL-12 modulated the expression of IL-21, IFN-γ and CD40L in purified polyp CD8^+^ T cells. Moreover, the percentage of IL-21^+^CD8^+^ T cells in polyp tissues was positively correlated with endoscopic and CT scan scores in NP patients. These findings indicated that polyp CD8^+^ T cells, by co-expressing IL-21 and IFN-γ and other markers, display a Tfh cell functionality, which is associated with the clinical severity of NP patients.

Nasal polyps (NPs) is a chronic inflammatory disease of the upper airway and commonly arise from the paranasal sinuses^1^. It remains one of the most challenging diseases in the field of rhinology due to its complex etiology and frequent recurrence. NPs is thought as a multifactorial disease with several different etiological factors, including infection and allergy^2^. T lymphocytes are critical for the development of sinonasal inflammation, both CD4^+^ T cells (including Th1, Th2, Th17 and Treg cells) and their cytokines have been reported to display different immune effects in the local inflammatory reaction of NPs^3^,^4^,^5^,^6^. Moreover, regional (i.e., Asian vs. Western countries) and/or racial differences in the roles of various Th cell subsets in the local inflammatory reaction in NP tissues have been reported^7^. Similar to the subsets of CD4^+^ T cells, the subsets of CD8^+^ T cells, including Tc1, Tc2, and Tc17 cells, and their interrelated cytokines have been shown to play an important role in the adaptive immune response to pathogens in various infectious and inflammatory diseases, including HIV infection^8^, rheumatic diseases^9^ and tuberculosis^10^. In airway diseases, CD8^+^ T cells were found to suppress allergen-induced late airway responses, inhibit airway eosinophilia through secretion of IFN-γ and mediate pulmonary alveolitis and inflammation^11^,^12^. However, the clinical significance of CD8^+^ T cells in NPs remains to be elucidated. Interleukin-21 (IL-21), a member of the common-γ chain (γc) family of cytokines, has the ability to act on multiple cells of the immune system^13^. Consistent with its broad effects, it has become clear that not only does IL-21 regulate normal lymphoid development and function, but it also serves critical roles in inflammatory, allergic, autoimmune and neoplastic diseases^14^. However, it is unknown whether IL-21 is involved in the sinonasal inflammatory responses of NP patients.

Our previous studies showed that polyp tissues contained significantly higher IL-21 production compared with control mucosa tissues and that IL-21 level positively correlated with polyp size and surgical recurrence of NP patients^15^. IL-21 is normally thought to be produced by various subsets of CD4^+^ T cells and NKT cells^16^. In the present study, we found that CD8^+^ T cells from polyp tissues produced high levels of IL-21 as well. Some of the IL-21-producing CD8^+^ T cells in polyp tissues co-expressed IFN-γ, CXCR5, PD-1, ICOS, T-bet and Bcl-6, which display the T follicular helper (Tfh) cell functionality. Moreover, IL-21-expressing CD8^+^ T cells were positively regulated by IL-12, and the percentages of IL-21-expressing CD8^+^ T cells were associated with the clinical severity of NP patients.

## Result

### Polyp CD8^+^ T cells expressed higher IL-21, IL-17A and IFN-γ levels than in control sinonasal tissues

**Figure 1:**
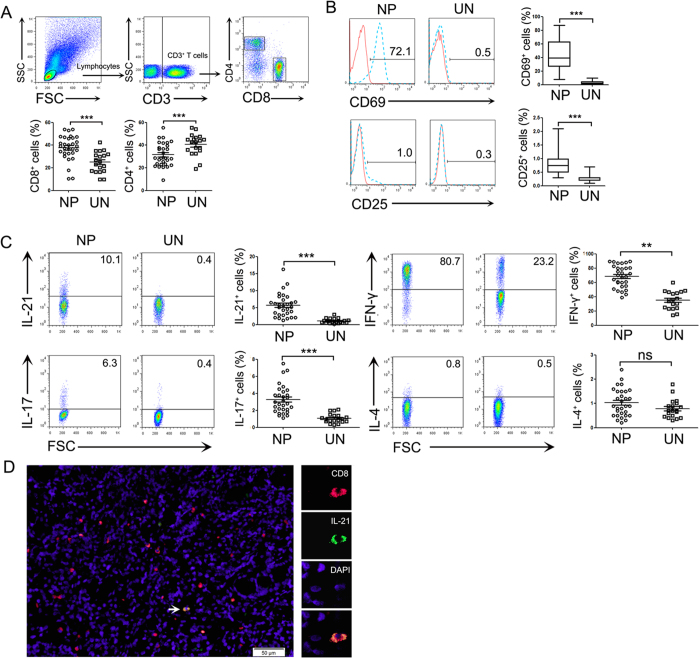
CD8^+^ T cells expressed higher IL-21, IL-17 and IFN-γ levels in polyp tissues than in control uncinate tissues. (**A**) FACS gating is shown for the CD8^+^ T cell analysis. Summary data are shown, which includes the percentage of CD8^+^ and CD4^+^ T cells in the CD3^+^ T cells from polyp (n = 30) and control uncinate tissues (n = 19). (**B**) Representative FACS data and statistical analyses shows the CD69 and CD25 expression levels on unstimulated CD8^+^ T cells from polyp (n = 30) and control uncinate tissues (n = 19). (**C**) Lymphocytes were analyzed by flow cytometry after stimulation with PMA/ionomycin and BFA for 5 h. Representative FACS data and statistical analysis showed the IL-21, IL-17, IFN-γ and IL-4 expression levels by CD8^+^ T cells from polyp (n = 25) and control uncinate tissues (n = 19). (**D**) Representative microphotographs of CD8 (red)-, IL-21 (green)- and DAPI (blue)-stained paraffin sections of polyp tissues (n = 12). The arrow displays the colocalization of CD8 and IL-21 (scale bars represent 50 μm). **P < 0.01; ***P < 0.0001; ns, no significance. NP, nasal polyp; UN, uncinate.

First, we analyzed the percentage of CD8^+^ and CD4^+^ T cells in polyp T lymphocytes by flow cytometry. The results showed that the proportion of CD8^+^ T cells in polyp tissues was significantly higher than in control tissues, and the percentage of CD4^+^ T cells in polyp tissues was less than in control sinonasal tissues (*P* < 0.01, Fig. 1A). In the absence of stimulation, the expression of CD69 and CD25 on CD8^+^ T cells in polyp tissues was significantly increased compared with control tissues (*P* < 0.05, Fig. 1B).

Next, we evaluated cytokine expression by CD8^+^ T cells in response to PMA and ionomycin. In addition to IL-17A and IFN-γ, we found that polyp CD8^+^ T cells expressed higher IL-21 levels (8.8 ± 2.4%) compared with CD8^+^ T cells from control tissues (0.45 ± 0.11%, *P* < 0.01, Fig. 1C). Moreover, an immunofluorescence assay confirmed the presence of IL-21-expressing CD8^+^ T cells in polyp tissues (Fig. 1D).

### The characteristics of IL-21-expressing CD8^+^ T cells in polyp tissues

**Figure 2:**
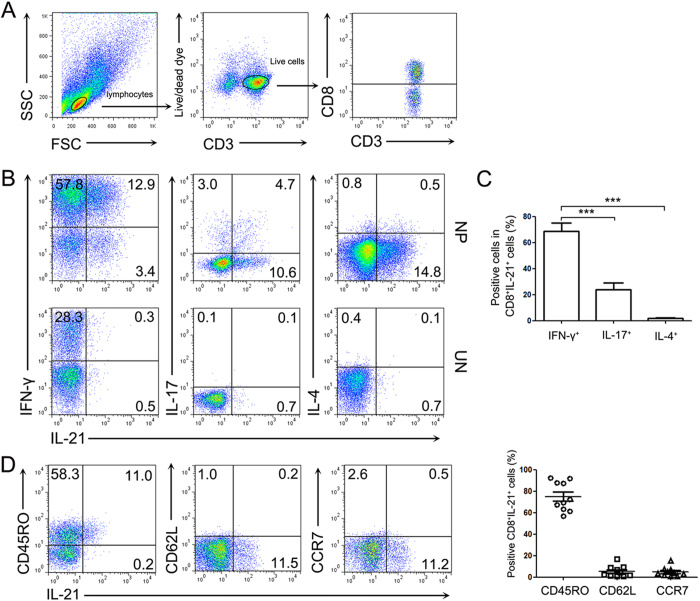
The characteristic of IL-21-expressing CD8^+^ T cells from polyp tissues. Lymphocytes were analyzed with flow cytometry after stimulation with PMA/ionomycin and BFA for 5 h. (**A**) FACS gating was used in the CD8^+^ T cell analysis. (**B**,**C**) Representative FACS data and summary analysis show the IFN-γ, IL-17 and IL-4 co-expression with IL-21 by CD8^+^ T cells from polyp (n = 30) and control uncinate tissues (n = 19). (**D**) Representative FACS data and summary results show the CD45RO, CD62L and CCR7 expression in IL-21-expressing CD8^+^ T cells from polyp tissues (n = 30). The statistical significance was determined with the Mann–Whitney U test. ***P < 0.001. NP, nasal polyp; UN, uncinate.

To evaluate the cell subsets of IL-21-expressing CD8^+^ T cells in polyp tissues, we compared the cytokine expression by CD8^+^ T cells in polyp and control sinonasal tissues. The results showed that the majority of IL-21-producing CD8^+^ T cells co-expressed IFN-γ (Tc1 cells) but not IL-17A (Tc17) or IL-4 (Tc2) (Fig. 2A,B). The percentage of IFN-γ^+^ cells amongst the IL-21^+^CD8^+^ T cells was significantly higher than the percentage of IL-17A^+^ and IL-4^+^ cells in the polyp tissues (*P* < 0.001, Fig. 2C). These data suggested that IL-21-producing CD8^+^ T cells were mainly Tc1 cells in polyp tissues.

To identify the phenotypic characteristics of IL-21-expressing CD8^+^ T cells in polyp tissues, we analyzed the expression of memory phenotype markers. Our results demonstrated that most IL-21^+^CD8^+^ T cells expressed high CD45RO levels but minimal CD62L and CCR7 levels (Fig. 2D), exhibiting an effector memory phenotype in polyp tissues.

### IL-21-producing CD8^+^ T cells in polyp tissues expressed CXCR5, PD-1 and ICOS

**Figure 3:**
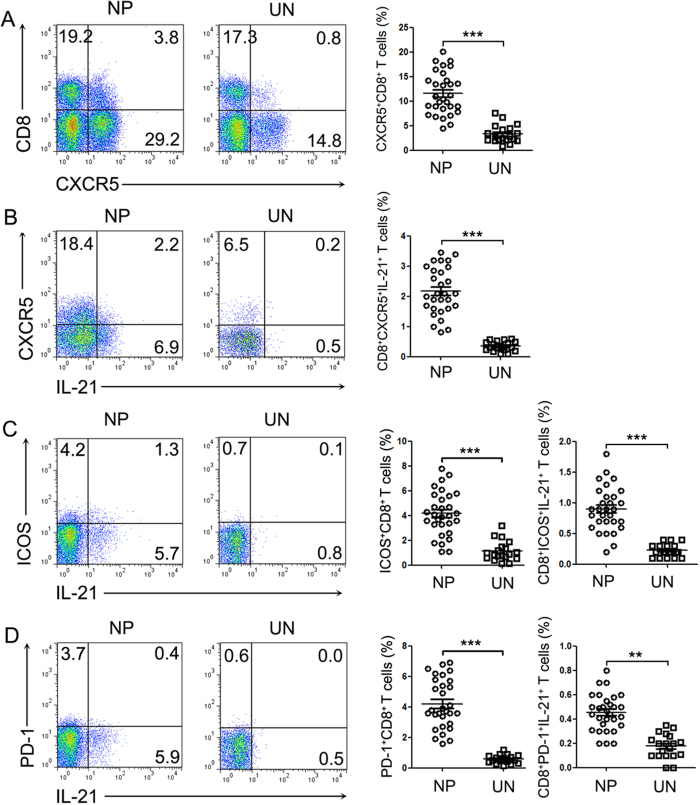
CD8^+^IL-21^+^ T cells from polyp tissues expressed CXCR5, ICOS and PD-1. (**A**) Representative FACS data and summary results show the CXCR5 expression levels on CD8^+^ T cells from polyp (n = 30) and control uncinate tissues (n = 19). (**B**–**D**) Lymphocytes were analyzed with flow cytometry after stimulation with PMA/ionomycin and BFA for 5 h. Representative FACS and summary results show the CXCR5, ICOS and PD-1 expression levels in IL-21^+^CD8^+^ T cells from polyp (n = 30) and control uncinate tissues (n = 19). **P < 0.01, ***P < 0.0001. NP, nasal polyp; UN, uncinate.

It has been reported that Tfh cells are a subset of CD4^+^ T cells and that they are distinguishable from Th1 and Th2 cells because they express IL-21, CXCR5, PD-1 and ICOS^17^. Here, we found that CXCR5, PD-1 and ICOS were also expressed on CD8^+^ T cells in polyp and control tissues in the absence of stimulation, which was similar to the Tfh cell phenotype. The expression of CXCR5, PD-1 and ICOS on CD8^+^ T cells in polyp tissues was significantly increased compared with control tissues (*P* < 0.01, Fig. 3), and some IL-21-expressing CD8^+^ T cells were found to co-express CXCR5, ICOS and PD-1 in polyp tissues. Moreover, the percentage of CXCR5^+^IL-21^+^, ICOS^+^IL-21^+^ and PD-1^+^IL-21^+^ cells in CD8^+^ T cells was higher in polyp tissues than in control sinonasal tissues (*P* < 0.01, Fig. 3). These results showed that IL-21^+^CD8^+^ T cells in polyp tissues, by co-expressing CXCR5, ICOS and PD-1, displayed the characteristics of Tfh cells.

### CD8^+^IL-21^+^ T cells highly expressed Bcl-6 and T-bet and promoted the production of IgG by B cells in polyp tissues

**Figure 4:**
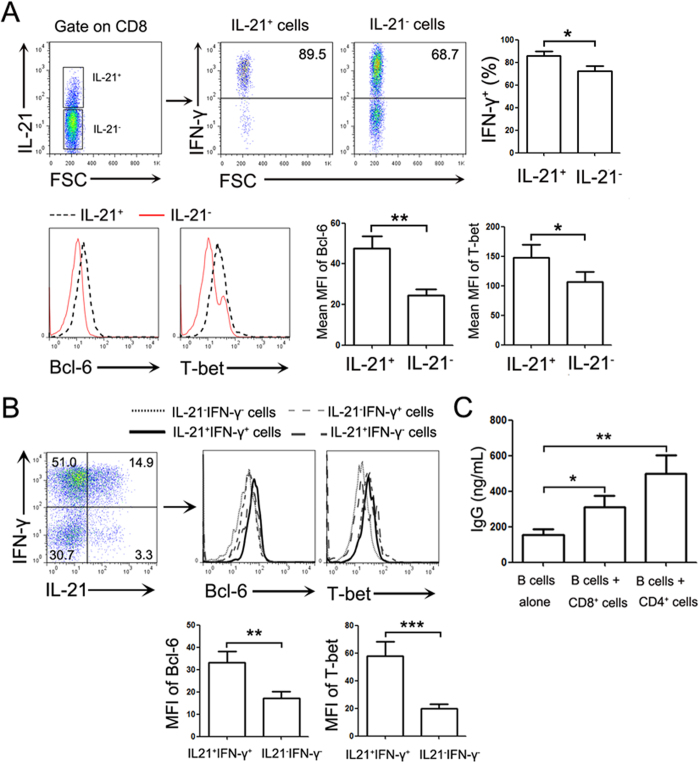
CD8^+^ T cells from polyp tissues highly expressed Bcl-6 and T-bet and promoted IgG production by B cells. (**A**) Representative FACS data and statistical analysis show the IFN-γ, Bcl-6 and T-bet expression levels by IL-21^+^ and IL-21^−^ cells among NP CD8^+^ T cells after stimulation with PMA and ionomycin. (**B**) The polyp CD8^+^ T cells were gated according to the IFN-γ and IL-21 expression after stimulation with PMA and ionomycin. Representative FACS and statistical data demonstrate the Bcl-6 and T-bet expression levels in each of the cell populations (n = 30). (**C**) CD19^+^ B cells were purified from polyp tissues and co-cultured with equal numbers of sorted CD8^+^ T cells, CD4^+^ T cells or B cells for 7 days. The IgG levels in the co-culture supernatant were determined by ELISA (n = 12). *P < 0.05; **P < 0.01; ***P < 0.001.

We further examined the expression of Bcl-6 (for IL-21) and T-bet (for IFN-γ) in IL-21-expressing CD8^+^ T cells from polyp tissues by flow cytometry. After stimulation with PMA and ionomycin, the IL-21^+^CD8^+^ T cells expressed significantly higher amounts of IFN-γ, Bcl-6 and T-bet than did the IL-21^−^CD8^+^ T cells (*P* < 0.05, Fig. 4A). CD8^+^ T cells were gated according to their IL-21 and IFN-γ expression levels. Compared with IFN-γ^−^IL-21^−^ T cells, IFN-γ^+^IL-21^+^ T cells showed higher Bcl-6 and T-bet expression levels (Fig. 4B).

To further confirm the biological function of CD8^+^ T cells on B cells, we sorted B cells and CD8^+^ T cells from polyp tissues. B cells were cultured over a period of 7 days either alone or with CD8^+^ T cells. A significant increase in IgG production by B cells was observed in the presence of CD8^+^ T cells compared with B cells alone (*P* < 0.01, Fig. 4C).

### IL-21 and IFN-γ co-expression by polyp CD8^+^ T cells was promoted by IL-12

**Figure 5:**
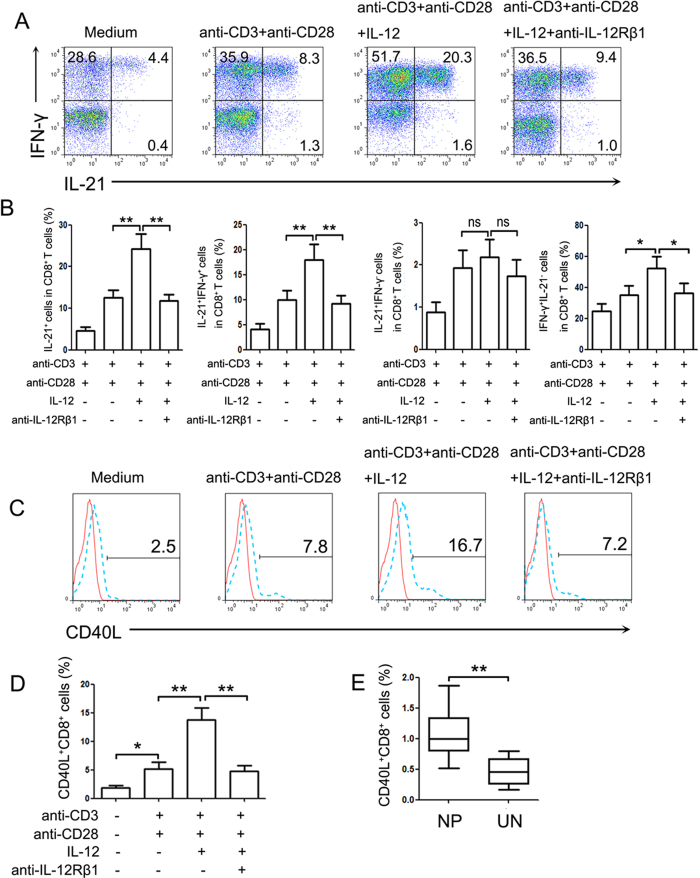
IL-12 upregulated the IL-21, IFN-γ and CD40L expression in CD8^+^ T cells from polyp tissues. Lymphocytes isolated from polyp tissues were stimulated with anti-CD3 and anti-CD28 in the presence or absence of IL-12 or anti-IL-12Rβ1 antibodies for 72 h. Then, the cells were harvested and re-stimulated with PMA and ionomycin in the last 5 h. CD8^+^ T cells were gated and assayed by FACS. (**A**) Representative data demonstrated the IL-21 and IFN-γ expression levels by CD8^+^ T cells (n = 18). (**B**) Summary results show the percentages of IL-21^+^, IL-21^+^IFN-γ^+^, IL-21^+^IFN-γ^−^ and IFN-γ^+^ IL-21^−^ cells in the CD8^+^ T cells. (**C**,**D**) Representative FACS graph and statistical data show the expression of CD40L on the observed CD8^+^ T cells (n = 18). (**E**) The summary data show the CD40L expression on unstimulated CD8^+^ T cells from polyp (n = 30) and control uncinate tissues (n = 19). *P < 0.05; **P < 0.01; ns, no significance. NP, nasal polyp; UN, uncinate.

Because others and our studies (data not shown) had found that IL-12 levels were significantly increased in polyp tissues compared with control sinonasal tissues, and IL-12 was previously found to induce the generation of IL-21 and IFN-γ co-expression by CD4^+^ T cells^15^, we evaluated whether IL-21-producing CD8^+^ T cells in NP tissues were regulated by IL-12. The isolated polyp lymphocytes were activated with anti-CD3 and anti-CD28 antibodies in the presence or absence of IL-12. The results showed that the addition of exogenous IL-12 to the cultures significantly increased the percentage of CD8^+^ T cells that were double-positive for IFN-γ and IL-21 and single-positive for IFN-γ (Fig. 5A,B, *P* < 0.05). Blockade of IL-12 with anti-IL-12Rβ1 antibodies inhibited the IL-12-induced increase in IL-21 and IFN-γ expression by CD8^+^ T cells. Additionally, we found that the expression of CD40L on freshly isolated and unstimulated polyp CD8^+^ T cells was higher than in cells from control sinonasal tissues (*P* < 0.01, Fig. 5E). IL-12 also significantly induced the expression of CD40 L on CD8^+^ T cells in polyp tissues (*P* < 0.01, Fig. 5C,D).

**Figure 6:**
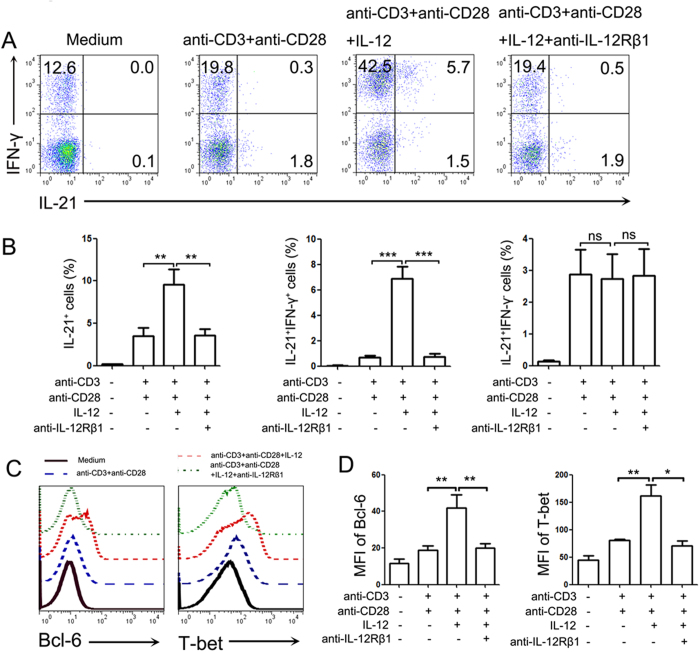
IL-12 directly regulated purified CD8^+^ T cells to express IL-21 and IFN-γ. Purified CD8^+^ T cells from PBMCs of NP patients were stimulated with anti-CD3 and anti-CD28 in the presence or absence of IL-12 and anti-IL-12Rβ1 antibodies for 72 h. PMA and ionomycin plus BFA were added in the last 5 h, and the cells were assayed with FACS. (**A**) Representative data show the IL-21 and IFN-γ expression levels by CD8^+^ T cells. (**B**) The statistical results show the percentage of IL-21^+^, IL-21^+^IFN-γ^+^ and IL-21^+^IFN-γ^−^ cells (n = 18) amongst the CD8^+^ T cells. (**C**,**D**) The representative FACS data and summary results show the Bcl-6 and T-bet expression levels by the CD8^+^ T cells (n = 18). *P < 0.05; **P < 0.01; ***P < 0.001; ns, no significance.

To further estimate the effect of IL-12 on the IL-21 production from CD8^+^ T cells, we purified CD8^+^ T cells from peripheral blood mononuclear cells (PBMCs) of NP patients and stimulated them with IL-12. The results showed that IL-12 significantly up-regulated the expression of IL-21 by purified CD8^+^ T cells, mainly by increasing the percentage of cells co-expressing IL-21 and IFN-γ (*P* < 0.01, Fig. 6A,B). Additionally, the Bcl-6 and T-bet expression in the purified polyp CD8^+^ T cells was increased after stimulation with IL-12 (*P* < 0.05, Fig. 6C,D).

### The clinical relevance of IL-21^+^CD8^+^ T cells in NP patients

**Figure 7:**
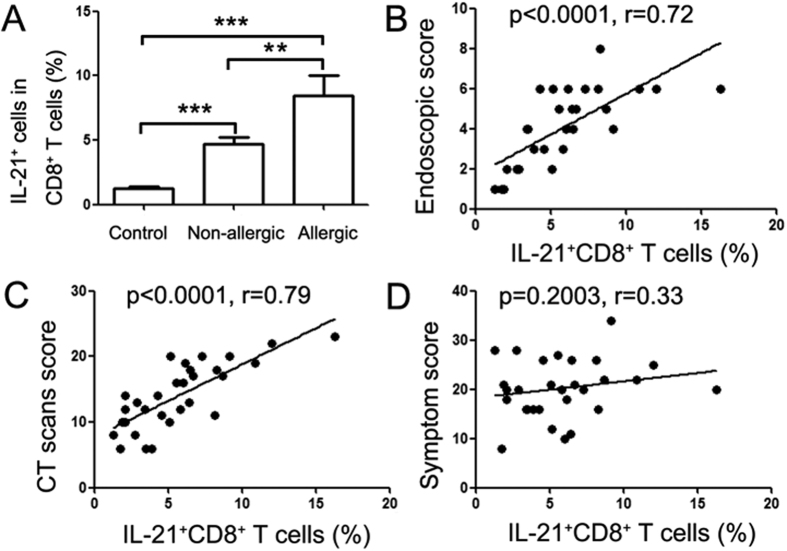
The percentage of IL-21^+^CD8^+^ T cells in polyp tissues was correlated with clinic characteristics of NP patients. Lymphocytes were analyzed with flow cytometry after stimulation with PMA/ionomycin and BFA for 5 h. (**A**) The summary data show the percentage of IL-21^+^ cells amongst the CD8^+^ T cells from non-atopic (n = 19) and atopic (n = 11) NP patients. *P < 0.05; ***P < 0.001. (**B**–**D**) Data show the correlation between the percentages of IL-21^+^CD8^+^ T cells in the polyp tissues and endoscopic, CT scan and symptom scores, as evaluated by VAS for NP patients (n = 30). Correlations were determined with the Spearman’s rank correlation coefficients.

To investigate the significance of IL-21-expressing CD8^+^ T cells in polypogenesis, we analyzed the association of polyp IL-21-expressing CD8^+^ T cells and the clinical features of NP patients. We found that the expression of IL-21 by CD8^+^ T cells, in polyp tissues of both atopic and non-atopic patients, was significantly increased compared with control sinonasal tissues. The percentage of IL-21^+^CD8^+^ T cells in the atopic NP patients was higher than in non-atopic NP patients (*P* < 0.01, Fig. 7A). Moreover, the IL-21-expressing CD8^+^ T cells in polyp tissues were positively correlated with the endoscopic and CT scores in NP patients (*P* < 0.01, Fig. 7B–D), suggesting that CD8^+^ T cells may play a critical role in the formation of NPs.

## Discussion

NPs is a chronic inflammatory condition of the nasal mucosa that is are characterized by infiltration of the mucosa with various inflammatory cells. T cells are well recognized to play an important role in the immune response of the local environment in sinonasal mucosa. Although some studies have demonstrated that the deviation of CD4^+^ and CD8^+^ T cells from normal levels was closely related with the pathology of chronic inflammatory disease^18^; whether CD4^+^ or CD8^+^ T cells are dominant in lymphocytes from polyp tissues has been debatable. Several experiments supported the finding that CD4^+^ T cells were dominant^19^, whereas other studies indicated that CD8^+^ T cells were in the majority in polyp tissues^20^,^21^. These different results may be due to polyp samples being obtained from varied regions and races in different studies^7^. In this study, we enrolled NP patients from southern China and found that these polyp tissues displayed a significant increase in CD8^+^ T cells and a relative deficiency of CD4^+^ T cells compared with control sinonasal tissues. The phenotypic analysis of polyp CD8^+^ T cells showed that the majority of the cells highly expressed CD69 and, to a lesser extent, CD25, and they were in an active state. The increased numbers and state of activation of CD8^+^ T cells suggested that they might be involved in the development of inflammatory responses in NP patients.

Prior evidence^22^, which was based on studies evaluating NP patients from Western countries, demonstrated that NPs was characterized by eosinophil infiltration and excessive Th2 cytokine expression. However, it is now recognized that polyp tissues from Chinese or Korean patients are biased toward neutrophilic inflammation and a significant increase in the Th1/Th17 cell pattern^7^. Our previous work also suggested that high percentages of Th1 and Th17 cells exist in polyp tissues in NP patients from southern China^3^. In the present study, we observed an increased percentage of IFN-γ^+^ (Tc1) and IL-17^+^ (Tc17) cells amongst the polyp CD8^+^ T cells, indicating a similarly mixed Tc1/Tc17 pattern.

It has been shown that IL-21 impacts many aspects of the immune-mediated control of infection^23^. It is widely recognized that IL-21 is preferentially produced by activated CD4^+^ T cells, including Tfh, Th17, and NKT cells^16^. However, the ability of CD8^+^ T cells to produce IL-21 has not yet been clearly elucidated. It had been demonstrated that IL-21 is produced by CD4^+^ but not CD8^+^ T cells in both Crohn’s disease and ulcerative colitis patients^24^. CD8^+^ T cells from HIV-1-infected individuals can produce IL-21, and IL-21-producing CD8^+^ T cells are associated with high activation levels^25^. In polyp tissues, we found that both CD4^+^ and CD8^+^ T cells produced IL-21 at higher levels than in control tissues^15^. The majority of IL-21-producing CD8^+^ T cells from polyp tissues co-expressed IFN-γ, but not IL-17 or IL-4, indicating that Tc1 cells were the main IL-21-producing CD8^+^ T cell subset in polyp tissues. These data suggested that, similar to CD4^+^ T cells, various CD8^+^ T cell subsets might acquire the ability to produce IL-21 depending on the presence of factors that may either positively or negatively regulate their activation. Additionally, we found that most of the IL-21-expressing CD8^+^ T cells were CD45RO^+^CCR7^−^CD62L^−^ T cells and that they displayed a terminally differentiated effector memory cell phenotype.

Tfh cells are characterized by the expression of IL-21, Bcl-6, CXCR5, and ICOS^26^ and provide help to B cells in lymphoid tissues. In the past decade, “unconventional” subsets of Tfh cells have also been identified, including extrafollicular Th cells, NKTfh cells, γδTfh cells, follicular Treg cells and circulating Tfh (CD4^+^CXCR5^+^) cells, which are called Tfh-like cells by displaying similar functionality^27^. Several studies^28^,^29^ reported the generation of Tfh-like cells by CD4^+^ T cells, which co-expressed both IL-21 and IFN-γ in the process of differentiation of Th1 cells. CD8^+^CXCR5^+^ T cells in tonsil B cell follicles^30^ could support survival and IgG production of tonsil B cells, suggesting that CXCR5^+^CD8^+^ T cells were part of the follicular T cell population. Similar to the findings that tonsil CD8^+^ T cells highly expressed CXCR5 (data not shown), we observed that CXCR5, ICOS and PD-1 expression was increased on CD8^+^ T cells and CD4^+^ T cells^15^ in polyp tissues compared with those in control tissues. Additionally, some polyp IL-21^+^CD8^+^ T cells were found to co-express CXCR5, ICOS or PD-1. The transcription factor analysis showed that IL-21-expressing CD8^+^ T cells in polyp tissues expressed higher levels of Bcl-6 and T-bet than did IL-21^−^CD8^+^ T cells, and polyp CD8^+^ T cells were capable of promoting IgG generation by purified polyp B cells. Though the molecular mechanism underlying IgG production in this co-culture system remains unclear, but we observed the IgG production induced by CD8^+^ T was both contact-dependent and IL-21-dependent (indicated by transwell cultured system or IL-21 antibody, data not shown). Taken together, we found that polyp CD8^+^ T cells displayed Tfh-like cell functionality.

Prior studies^28^,^29^ revealed that IL-12 can induce the generation of IL-21- and IFN-γ-producing CD4^+^ T cells, sharing the functional features of both Tfh and Th1 cells. Moreover, we (data not shown) and other groups^31^ also showed that IL-12 levels were increased in polyp tissues compared with control tissues. To evaluate whether IL-12 was able to increase IL-21 production by CD8^+^ T cells, we stimulated lymphocytes from polyp tissues or purified CD8^+^ T cellsfrom PBMCs of NPs patients with IL-12. The results demonstrated that IL-12 stimulation significantly enhanced the percentage of CD8^+^ T cells that expressed either IFN-γ or IL-21. Notably, IL-12 dramatically increased the percentage of CD8^+^ T cells that co-expressed IL-21 and IFN-γ but did not significantly alter the percentage of IL-21^+^IFN-γ^−^ CD8^+^ T cells. Simultaneously, we observed that the T-bet and Bcl-6 expression levels in CD8^+^ T cells were significantly up-regulated by IL-12.

CD40L expression by CD8^+^ T cells has been reported in selected experimental conditions^32^, and CD40L-expressing CD8^+^ T cells were found to induce IgE synthesis by normal human B cells in the presence of IL-4^33^. On the other hand, IL-12 is known to enhance CD40L expression in CD8^+^ T cells after differentiation and reactivation^34^. In the present study, our results showed that polyp CD8^+^ T cells expressed higher CD40L levels than did cells that were isolated from control tissues. Moreover, IL-12 stimulated significant CD40L expression by polyp CD8^+^ T cells, indicating that CD8^+^ T cells may provide help to B cells through the binding of CD40L and CD40 and participate in the development of inflammatory responses in NP patients. CD8^+^ T cells are multifunctional inflammatory cells that can produce various cytokines, and previous studies^35^ have shown that IFN-γ or IL-17 production by CD8^+^ T cells are involved in the inflammatory response. However, the clinical association of CD8^+^ T cells and NPs remained unclear. In the present study, our results showed that polyp CD8^+^ T cells produced high IL-21 levels, and the percentage of IL-21^+^CD8^+^ T cells in polyp tissues was positively correlated with the endoscopic and CT scan scores of NP patients. Thus, we provided evidence that CD8^+^ T cells may promote polypogenesis in an IL-21-associated manner. However, further study is still required to clarify the underlying molecular mechanism.

Taken together, our study showed that activated CD8^+^ T cells were predominant amongst the polyp lymphocytes and that they expressed high IL-21 levels, which was regulated by IL-12. IL-21-producing CD8^+^ T cells in polyp tissues, which co-expressed CXCR5, PD-1 and ICOS, displayed Tfh cell functionality by promoting IgG production in isolated polyp B cells, and the percentages of IL-21-producing CD8^+^ T cells were found to be associated with both the CT and endoscopic scores of NP patients. These findings, together with the demonstration that IL-21 drives various inflammatory pathways, reinforce the concept that CD8^+^ T cells may play a critical role in polypogenesis in an IL-21-associated manner.

## Materials and Methods

The study has been approved by Zhongshan School of Medicine, Guangdong General Hospital, Guangzhou First People’s Hospital and the First Affiliated Hospital of Sun Yat-sen University. All methods used in this study were carried out in accordance with the approved guidelines and all experimental protocols were approved by Institute of Immunology, Zhongshan School of Medicine of Sun Yat-sen University.

### Patients

Polyp specimens were obtained from 79 NP patients from the included hospitals between September 2013 and October 2015. Sixty polyp tissue specimens were obtained during routine functional endoscopic sinus surgery. The NP diagnoses were made according to the European Position Paper on Rhinosinusitis and Nasal Polyps 2012 criteria (EPOS 2012) and were based on patient history, clinical examination, nasal endoscopy and sinus computed tomography (CT) scanning. During the same time period, 18 peripheral blood samples were obtained from the NP patients. The atopic status of the subjects was evaluated with a skin-prick test to a panel of aeroallergens (e.g., pollens, dust mites, pets, and molds, etc.). Patients with an established immunodeficiency, pregnancy, classic allergic fungal sinusitis diagnosis or cystic fibrosis were excluded from this study. Nineteen control uncinate samples were collected as healthy control sinonasal tissues, which were obtained from individuals underwent septoplasty for anatomic variations during septal surgery, and the atopic patients were excluded. None of the subjects used oral or intranasal steroids for at least 2 weeks before sample collection. The study was approved by the Ethical Committee of the included hospitals and all the subjects signed informed consent forms. The detailed patient information is summarized in Table 1.

The Lund-Kennedy scoring system (0–6) was used to grade the polyp size, as follows: 0, no polyps; 1, polyps in the middle meatus but not reaching below the inferior border of the middle turbinate; 2, polyps reaching below the inferior border of the middle turbinate but not to the inferior border of the inferior turbinate; and 3, extensive large polyps congesting the inferior meatus. The CT scans were graded according to the Lund-MacKay method. The individual nasal symptom scores included nasal congestion, anterior rhinorrhea, postnasal drip and loss of smell, and they were evaluated with a visual analogue scale (VAS) system before surgery. The reasons for the surgical procedures were unrelated to the study in all of the patients.

### Cell isolation

Tissue samples were cut into small pieces and digested with endotoxin-free collagenase I (2 mg/ml, Sigma-Aldrich, St Louis, MO, USA) in incomplete RPMI-1640 for 1 h at 37 °C. Single cell suspensions were obtained by filtering through a 100-μm nylon mesh (BD Bioscience Pharmingen, San Diego, CA, USA). The mononuclear cells in the polyp and control tissues were isolated with Ficoll-Hypaque (Tianjin Hao Yang Biological Manufacture, Tianjin, China) density gradient centrifugation.

PBMCs were prepared with Ficoll-Hypaque density gradient centrifugation from the peripheral blood of NP patients. CD8^+^ T cells were positively purified from freshly isolated PBMCs with anti-CD8 microbeads (Miltenyi Biotec, Bergish Gladbach, Germany). B cells, CD8^+^ T cells and CD4^+^ T cells were sorted from polyp tissues using a FACS Aria II cytometer (BD company, San Jose, CA, USA). The purity of cells exceeded 94%.

### Cell culture conditions

To determine the cytokine and transcription factor expression levels, the lymphocytes that were isolated from the polyp and control sinonasal tissues were stimulated for 5 h with PMA (20 ng/ml; Sigma-Aldrich) and ionomycin (1 μg/ml; Sigma-Aldrich) at 37 °C with 5% CO_2_ in the presence of brefeldin A (10 μg/ml; Sigma-Aldrich).

In some experiments, lymphocytes that were isolated from polyp tissues or purified CD8^+^ T cells from PBMCs were stimulated with immobilized anti-CD3 (1 μg/ml; BD Bioscience PharMingen) and anti-CD28 (1 μg/ml; BD Bioscience PharMingen) in the presence or absence of IL-12 (5 ng/ml, eBioscience, Santiago, Chile) or anti-IL-12Rβ1 antibodies (10 μg/ml, Hoffmann-La Roche Inc, USA) for 72 h. The cell-free supernatants were harvested and assayed by ELISA for the production of IL-21 or IFN-γ. The cells were collected and stimulated for 5 h with PMA, ionomycin and BFA. The IL-21 and IFN-γ expression levels were assayed by flow cytometry.

### ELISA

ELISA was performed according to the manufacturer’s instruction. The detection limits were as follows: 31 pg/mL for IL-21 (eBioscience) and 4.7 pg/mL for IFN-γ (BD Bioscience Pharmingen). For convenient analysis, all of the values that were less than the detectable limit were considered to be zero.

### Flow cytometry

Before staining, cells were incubated in green fluorescent reactive dye (Invitrogen Life Technologics, Carlsbad Calif) for 30 minutes for dead cell discrimination. The cells were washed twice with PBS buffer containing 0.1% BSA and 0.05% sodium azide. For surface staining, cells were incubated with the respective mAbs at 4 °C in the dark for 30 min. For the detection of intracellular cytokines, cells were fixed with 4% paraformaldehyde and permeabilized in PBS buffer containing 0.1% saponin (Sigma-Aldrich), 0.1% BSA and 0.05% NaN_3_ for at least 2 h or overnight at 4 °C and stained with conjugated mAbs for intracellular cytokines. For the intracellular transcription factor detection, cells were stained for surface antigens, followed by fixation and permeabilization with Permeabilization/Fixation buffer (BD Bioscience PharMingen) and they were stained according to the Permeabilization/Fixation Kit protocol. The stained cells were washed twice before analysis using a FACS Aria II cytometer (BD company, San Jose, CA, USA). Lymphocytes were gated on forward and side scatter profiles and analyzed using the FlowJo software (Treestar, San Carlos, CA, USA).

The mAbs were used for cell surface or intracellular staining, isotype-matched control antibodies, purified anti-CD3 and anti-CD28 mAb were purchased from BD Bioscience Pharmingen.

### Immunofluorescence assay

Paraffin sections of the polyp tissues were rehydrated and boiled in EDTA buffer (pH8.0) for 10 min to induce antigen retrieval. After washing, the tissue sections were blocked for nonspecific binding with 5% goat serum/0.3% Tween-20/PBS, and incubated with rabbit anti-human IL-21 (abcam, polyclone, 1:100) and mouse anti-human CD8 antibodies (abcam, clone: 144B, 1:200) at 4 °C overnight. After washing, the sections were incubated with Alexa Fluor 488–conjugated goat anti-mouse IgG (Invitrogen, 1:1000) and Alexa Fluor 555–conjugated goat anti-rabbit IgG (Invitrogen, 1:1000) for 2 h at room temperature in the dark. After a final washing, the cover slips were mounted onto slides with fluoroshield mounting medium and DAPI (4′,6-diamidino-2-phenylindole; abcam). The slides were observed with an Olympus BX53 microscope, and the images were collected with the Cell Sense software (Olympus, Center Valley, Pa).

### Statistical analysis

The tissue data are expressed as medians (range). Comparisons between two groups were performed with the non-parametric Mann–Whitney *U*-test. For these assays, the data are expressed as the mean ± SEM. Comparisons between multiple groups were performed using the one-way analysis of variance (ANOVA) test followed by Student’s t-test. Correlations between variables were determined with Spearman’s rank correlation coefficients. *P* < 0.05 was considered to be statistically significant.

## Additional Information

**How to cite this article**: Xiao, L. *et al.* Phenotypic and functional characteristics of IL-21-expressing CD8^+^ T cells in human nasal polyps. *Sci. Rep.* **6**, 30362; doi: 10.1038/srep30362 (2016).

## Figures and Tables

**Table 1:** The characteristics of subjects.

Characteristics	NPs patients	Normal controls
Subject no.	60	19
Gender (M/F)	38/22	12/7
Age (range)	46 (13~74)	38 (27~43)
Atopy	11	0
Asthma history	4	0
Aspirin intolerance	0	0
CT score	18.2 (5~24)	0
Nasal endoscopy score	4 (1~6)	0
Sinus surgery history	8	0
Methodologies used		
Flow cytometry	30	19
IHC	12	0
*In vitro* cell culture assay	30	0
